# Transcriptome analysis of the two unrelated fungal β-lactam producers *Acremonium chrysogenum* and *Penicillium chrysogenum*: Velvet-regulated genes are major targets during conventional strain improvement programs

**DOI:** 10.1186/s12864-017-3663-0

**Published:** 2017-03-31

**Authors:** Dominik Terfehr, Tim A. Dahlmann, Ulrich Kück

**Affiliations:** Lehrstuhl für Allgemeine und Molekulare Botanik, Ruhr-University Bochum, Universitätsstr. 150, Bochum, 44780 Germany

**Keywords:** Penicillin, Cephalosporin, Gene expression, *Acremonium chrysogenum*, *Penicillium chrysogenum*, Velvet, RNA-seq, Strain improvement, Secondary metabolism, Amino acid metabolism

## Abstract

**Background:**

Cephalosporins and penicillins are the most frequently used β-lactam antibiotics for the treatment of human infections worldwide. The main industrial producers of these antibiotics are *Acremonium chrysogenum* and *Penicillium chrysogenum,* two taxonomically unrelated fungi. Both were subjects of long-term strain development programs to reach economically relevant antibiotic titers. It is so far unknown, whether equivalent changes in gene expression lead to elevated antibiotic titers in production strains.

**Results:**

Using the sequence of PcbC, a key enzyme of β-lactam antibiotic biosynthesis, from eighteen different pro- and eukaryotic microorganisms, we have constructed a phylogenetic tree to demonstrate the distant relationship of both fungal producers. To address the question whether both fungi have undergone similar genetic adaptions, we have performed a comparative gene expression analysis of wild-type and production strains. We found that strain improvement is associated with the remodeling of the transcriptional landscape in both fungi. In *P. chrysogenum*, 748 genes showed differential expression, while 1572 genes from *A. chrysogenum* are differentially expressed in the industrial strain. Common in both fungi is the upregulation of genes belonging to primary and secondary metabolism, notably those involved in precursor supply for β-lactam production. Other genes not essential for β-lactam production are downregulated with a preference for those responsible for transport processes or biosynthesis of other secondary metabolites. Transcriptional regulation was shown to be an important parameter during strain improvement in different organisms. We therefore investigated deletion strains of the major transcriptional regulator *velvet* from both production strains.

We identified 567 *P. chrysogenum* and 412 *A. chrysogenum* Velvet target genes. In both deletion strains, approximately 50% of all secondary metabolite cluster genes are differentially regulated, including β-lactam biosynthesis genes. Most importantly, 35-57% of Velvet target genes are among those that showed differential expression in both improved industrial strains.

**Conclusions:**

The major finding of our comparative transcriptome analysis is that strain improvement programs in two unrelated fungal β-lactam antibiotic producers alter the expression of target genes of Velvet, a global regulator of secondary metabolism. From these results, we conclude that regulatory alterations are crucial contributing factors for improved β-lactam antibiotic titers during strain improvement in both fungi.

**Electronic supplementary material:**

The online version of this article (doi:10.1186/s12864-017-3663-0) contains supplementary material, which is available to authorized users.

## Background

In the last century, antibiotics have revolutionized the treatment of bacterial infections. β-lactam antibiotics represent a large fraction of these drugs, and are the product of bacterial or fungal secondary metabolism. The first two steps in β-lactam biosynthesis are identical in all pro- and eukaryotic microbes. In the first catalytic step, δ-(L-α-aminoadipyl)-L-cysteinyl-D-valine (ACV) synthetase (PcbAB) condenses L-cysteine, L-valine and L-α-aminoadipate to the linear ACV tripeptide, which is then converted in a second step by isopenicillin N synthase (PcbC) into the bicyclic intermediate isopenicillin N. This is the first intermediate of penicillin biosynthesis with antibacterial activity, and contains the β-lactam-ring system, for which this family of drugs is named. All further downstream reactions are distinct and species-specific, resulting in a variety of different β-lactam antibiotics including cephamycins, cephalosporins and penicillins [1–4]. *pcbAB* and *pcbC,* the genes coding for the first two enzymes involved in β-lactam biosynthesis, are genomically clustered in both bacteria and fungi. In the latter, the organization is identical in that both genes share a common promoter sequence. This clustered organization and the lack of intronic sequences in most of the genes led to the assumption that bacterial β-lactam antibiotic genes were transferred horizontally from bacteria to fungi [5].

In the treatment of human infections, the two most potent β-lactam antibiotics are penicillin and cephalosporin, which are synthesized by two filamentous fungi *P. chrysogenum* and *A. chrysogenum*. These two ascomycete species are only slightly taxonomically related. While *P. chrysogenum*, like *Aspergillus nidulans*, belongs to the order of Eurotiales, *Acremonium* species belong to the order of Hypocreales, which also incorporates *Claviceps*, *Fusarium* and *Trichoderma* species. Although *P. chrysogenum* was recently re-identified as *Penicillium rubens* [6, 7], we refer to all isolates as *P. chrysogenum* given that this is *nomen conservandum* [8].

Despite the fact that *A. chrysogenum* and *P. chrysogenum* are only distantly related, what both have in common is that they were subjects of intensive strain improvement programs including multiple rounds of random mutagenesis steps to increase the β-lactam antibiotic titer. This is based on the assumption that mutations detectable at the DNA sequence level are responsible for high antibiotic titers in production strains. So far, genomic comparisons between fungal wild-type and production strains were only performed for *P. chrysogenum* [9, 10]. This work detected mainly single nucleotide polymorphisms (SNPs), which let the authors conclude that expressional changes are largely responsible for observed enhanced penicillin titers. In line of this conclusion, transcriptional expressional studies were conducted recently with wild-type and production strains indicating a complex transcriptome regulation during fermentation in high yield strains [11]. Here, we addressed the question of whether β-lactam biosynthesis in both fungi has undergone parallel changes in global gene expression during strain improvement programs. Therefore, we will give a detailed description of strains used in this investigation.

After Fleming’s discovery of the antibacterial action of penicillin in 1928, strain development projects were initiated to increase penicillin production to meet the steadily rising demand for medical purposes [12, 13]. During global screening for penicillin-producing strains at the Northern Regional Research Laboratory (Peoria, Illinois, USA) strain NRRL 1951 was selected, which is the ancestral strain for all β-lactam production strains used in industrial applications. Based on NRRL 1951 (ATCC 9480), which is used in this investigation, derived production strains such as Q176 were obtained by X-ray, UV, or chemical mutagenesis [14]. All industrial strains used today are derivatives of this lineage. This includes Wisconsin 54–1255 for which the genome was recently determined [15], and P2 (ATCC 48271), another independently derived producer strain [16, 17]. The latter has an 85-fold increase in penicillin titer compared to NRRL 1951; P2 was used for further conventional mutagenesis to construct the nitrate reductase-deficient variant P2niaD18 [18–20]. This strain is used in this study for further analysis because it is genetically well-characterized and was the subject of recent genome and small RNA analysis [20, 21].

*Acremonium chrysogenum*, the second β-lactam-producing fungus analyzed in this study, was first isolated in 1945 from Sardinian coastal seawater by Guiseppe Brotzu. A few years later; in 1955, the antibiotic-acting secondary metabolite was isolated and characterized [22]. The outstanding differences between cephalosporin C and penicillins are its broad-spectrum activity against Gram-positive and -negative bacteria and resistance against penicillinases. Because of these favorable characteristics, strain improvement programs were initiated in the 1950s using random mutagenesis originating from Brotzus isolate (ATCC 11550). This first rounds of strain improvement at the Antibiotic Research Center in Clevedon (United Kingdom) resulted in the selection of an improved strain, M-8650, which is the progenitor strain of many industrial programs [23]. This early producer was able to synthesize 0.3 g/L of cephalosporin C [24]. So far, little information is available about the subsequent strain improvement programs. As compared to the M-8650 strain, A3/2 is a commonly used producer with an up to 100-fold increased production capacity [25]. This strain stems from a lineage of industrial production strains and has recently been used in numerous molecular genetic studies [26–28].

The strain improvement process via random mutagenesis has led to enhanced production titers of penicillin and cephalosporin C. As the name ‘random mutagenesis’ suggests, most of the changes at the genome level resulting in genome rearrangement and differential gene expression are unknown. Genome rearrangements in production strains were recently reported for both Ascomycetes. However, changes at the genome level did not explain the high titers of β-lactam antibiotics in these strains. For *P. chrysogenum*, it was proposed that differential gene expression rather than point mutation have led to the improved yields of industrial strains [10]. Therefore, we sought to elucidate global transcriptional changes that occurred during strain improvement of these two distantly related β-lactam producers. We performed transcriptional profiling on a genomic scale of wild-type and production strains from *P. chrysogenum* and *A. chrysogenum*.

As mentioned above, fungi have likely obtained β-lactam biosynthesis genes through horizontal gene transfer from bacteria. This process has further consequences for regulating expression of these bacterial genes in a eukaryotic host. Prokaryotic genes must be adapted to the eukaryotic host system and its expression machinery. While prokaryotic pathway-specific regulators commonly determine gene expression, several global regulators govern eukaryotic gene expression of secondary biosynthesis clusters. A well-studied example is the fungal Velvet multi-subunit complex, a key regulator of fungal development, which in recent years was shown to affect secondary metabolism in a wide range of diverse filamentous fungi. For *P. chrysogenum* and *A. chrysogenum,* we have previously shown that mutants lacking the *velvet* gene have significantly reduced β-lactam antibiotic biosynthesis [29–32].

Here, we addressed the question whether both distantly-related organisms have undergone similar genetic adaptations leading to economically relevant β-lactam antibiotic production levels. We analyzed the transcriptomes of wild-type and production strains by RNA-seq and searched for genes that are differentially regulated in both species. Because transcriptional regulation appears to be an important parameter, we analyzed mutants from both species lacking the *velvet* gene for the core protein of the Velvet complex [33–35]. We believe that our data will promote rational strategies for the construction of advanced industrial strains.

## Results and discussion

### Occurrence of β-lactam biosynthesis genes in eukaryotes

**Fig. 1:**
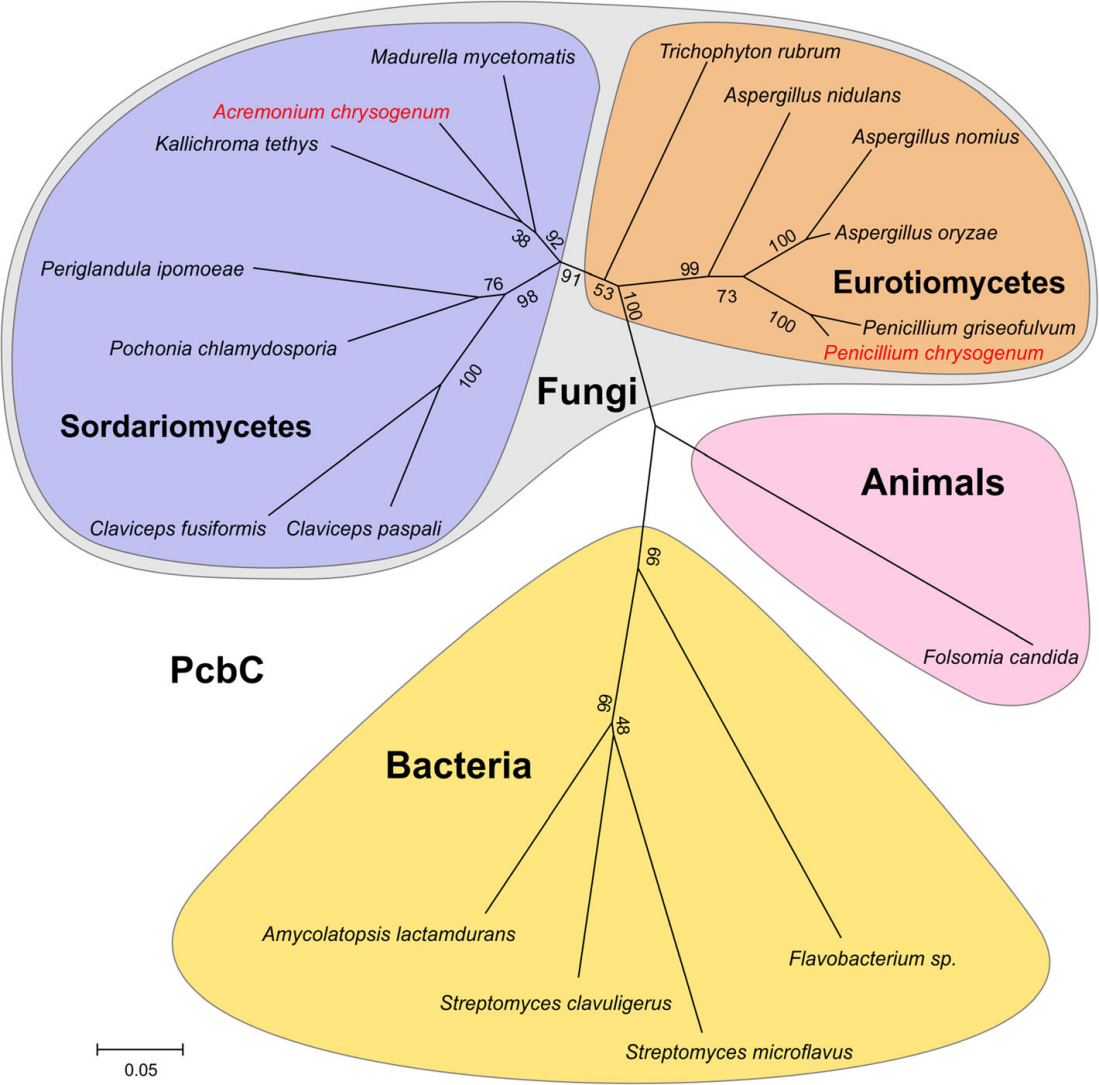
Phylogenetic tree of PcbC. The tree displays the relationship of a variety of species carrying orthologous *pcbC* genes. Amino acid sequences were taken from the NCBI database. A bootstrap of 1000 repetitions using the ‘neighbor-joining’ grouping method was performed; Numbers give bootstrap values of the corresponding nodes. Separation of *pcbC* carrying fungi in the two distantly related classes of Sordariomycetes and Eurotiomycetes is shown

In order to demonstrate the relationship between both taxonomically unrelated fungi, a key enzyme of β-lactam biosynthesis, which occurs in pro- as well as in eukaryotic organisms, was chosen for a phylogenetic analysis. β-lactam biosynthesis genes in fungal and bacterial genomes display similar gene organizations. In bacteria, a single gene cluster carries the essential genes for β-lactam biosynthesis, including *pcbAB*, *pcbC*, *cefE*, *cefF*, and *cefD* [36]*,* while two separate gene clusters exist in filamentous fungi [3]. The clustered *pcbAB* and *pcbC* are present within the genomes of members of Sordariomycetes and Eurotiomycetes [37]. *pcbC*-encoded isopenicillin N synthase (IPN) is common to all β-lactam producing organisms, and its amino acid sequence was used for a comparative phylogenetic analysis to enlighten the evolution of the early penicillin gene cluster in β-lactam antibiotic-producing organisms. For comparison, the amino acid sequence of IPN from *P. chrysogenum* was used to search public sequence databases, including the whole-genome sequence database available from the National Center for Biotechnology Information (NCBI). This allowed us to integrate recently sequenced but not yet annotated genome sequences. We identified *pcbC* homologues with a minimal identity of at least 75% in various bacterial species, mostly members of Streptomycetes, and in several fungal species, including members of Sordariomycetes and Eurotiomycetes. As distinct examples, we include *A. chrysogenum*, *Kallichroma tethys*, *Madurella mycetomatis*, and *Pochinia chlamydosporia* from Sordariamycetes in our tree, while we have chosen only a few members of the Eurotiomycetes. These are *Aspergillus nidulans*, *Aspergillus nomius*, *Aspergillus oryzae*, *P. chrysogenum*, *Penicillium griseofulvum*, and *Trichophyton tonsurans.* Within Eurotiomycetes all *pcbC*-containing fungi are either members of Eurotiales or Onygenales. Interestingly, in the latter group, *pcbC* is found in skin-infecting dermatophytes of the genus *Arthroderma* or its related anamorph *Trichophyton*. Within the Sordariomycetes, only a single fungal species outside of Hypocreales contains a *pcbC* orthologue: *M. mycetomatis*. It is the causal agent of the chronic granulomatous skin-disease eumycetoma and was recently classified as a member of the order Sordariales [38, 39]. Furthermore, genes with lower identity (58-71%) were detected in *Claviceps spec.* that carry complete open reading frames. We further identified a putative incomplete *pcbC*-like pseudogene in the *Periglandula ipomoeae* genome. We detected a nonsense mutation at codon 197 leading to a premature stop codon. Phylogenetic analyses based on identified PcbC amino acid sequences (Additional file 1: Figure S1) resulted in the construction of a phylogenetic tree reflecting the taxonomic classification of their suppliers (Fig. 1). Bacterial and fungal PcbC-like proteins are clearly separated. Within the fungi, distinct separation between Sordariomycetes and Eurotiomycetes illustrate the distant taxonomic relationship of *A. chrysogenum* and *P. chrysogenum. Penicillium* species containing *pcbC* are grouped with taxonomically closely-related *Aspergillus* species. As mentioned above, only one wild-type strain of *A. chrysogenum* is currently available, which was isolated from Sardinian coastal seawater. Interestingly, the most closely-related fungus carrying *pcbC* found in our analysis is the marine fungus *K. tethys* [40]. The first and thus far only functional IPN found in the animal kingdom was recently described in the terrestrial springtail *Folsomia candida* [41]. This *pcbC* homologue has a completely different gene structure with several introns that are absent from the microbial examples mentioned above. The animal *pcbC* homologue is clearly separated from all other *pcbC*-like genes in the phylogenetic tree.

Our phylogenetic analysis illustrates the distribution of *pcbC*-like genes within the different kingdoms, with an emphasis to fungi. These results underline the phylogenetic distance between the two fungal species of this investigation.

### Strain improvement is correlated to transcriptional landscape remodeling in *P. chrysogenum* and *A. chrysogenum*

In order to address the question whether both unrelated fungi have undergone similar genetic adaptions, a comparative gene expression analysis was performed using *P. chrysogenum* NRRL 1951 and P2niaD18 as well as *A. chrysogenum* ATCC 11550 and A3/2. RNA-seq data for each strain were obtained from two independent biological replicates and analysis was performed using DEseq2 [42]. We chose culture conditions that were previously found to be optimal for measuring the activity of β-lactam antibiotic biosynthesis genes [26, 30, 43–45]. Therefore, we grew all strains for three days in shaking flasks with rich Complete Culture Medium (CCM). This time point represents the early phase of β-lactam production and is characterized by an expressional switch from genes related to vegetative growth to those involved in secondary metabolite formation [46].

**Fig. 2:**
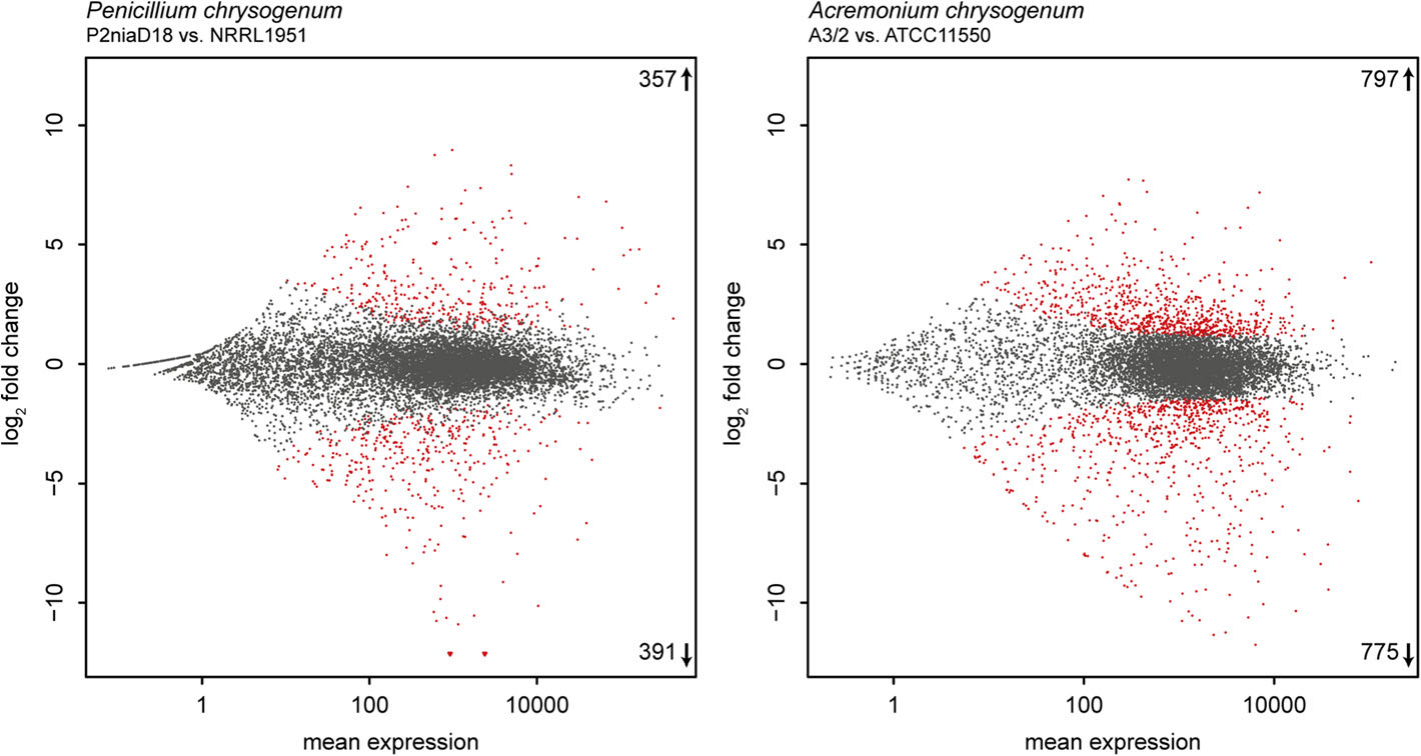
Differential gene expression in industrial production strains. MA-plots comparing gene expression data from industrial production strains of *P. chrysogenum* and *A. chrysogenum* compared to the corresponding wild-type strains. Log_2_-fold change as well as the mean expression from two replicates are shown. *Red* dots represent significantly differentially expressed genes, while grey dots mark similarly expressed genes

For *P. chrysogenum,* we identified 748 genes showing differential expression in P2niaD18 as compared to its ancestral wild type. This corresponds to 6.7% of the annotated protein-coding genes. Within differentially expressed genes, 357 (47.7%) show increased and 391 (52.3%) decreased expression profiles (Fig. 2). For *A. chrysogenum*, we detected 1572 differentially expressed genes in the industrial strain A3/2, which constitutes 17% of the annotated genes. From these, 797 (50.7%) were up- and 775 (49.3%) were downregulated in the industrial as compared to the wild-type strain. A summary of similar regulated orthologous genes in both industrial strains is provided in Additional file 2: Table S1.

We next performed a functional categorization with FungiFun2 [47] using the FunCat catalogs of *P. chrysogenum* and *A. chrysogenum* [48]. A FunCat catalog contains functional descriptions of proteins summarized in 28 main functional categories (first level). Each of these main categories comprises up to six levels in a hierarchical structure and an increasing level of specificity. Statistically overrepresented functional categories were determined for both, up- and downregulated genes. We focused our investigation on differentially expressed genes that have a direct or indirect impact on β-lactam antibiotic biosynthesis. Functional categories related to the main categories ‘metabolism,’ ‘cellular transport’ and ‘cell rescue, defense and virulence’ were significantly enriched in production strains of both filamentous fungi, whereas enrichment of the categories ‘binding’ and ‘energy’ were only significantly different in *A. chrysogenum*.

### Genes involved in primary and secondary metabolism are differentially expressed in industrial strains

**Fig. 3:**
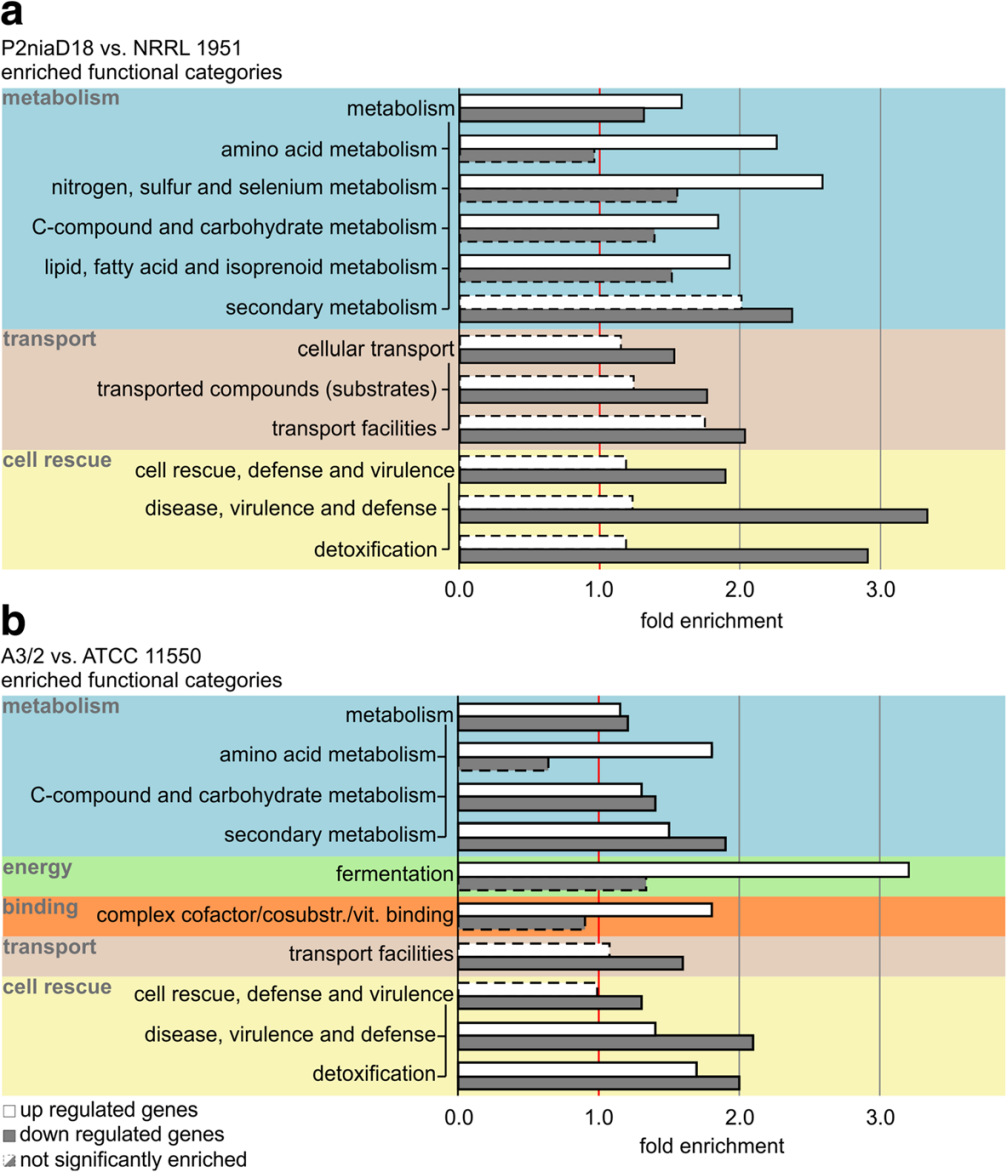
FungiFun2 enrichment analysis and functional categorization (FunCat) of differentially expressed genes in industrial strains. Industrial strains were compared with the corresponding wild-type strains. **a** *P. chrysogenum*: Within the comparative data set of P2niaD18 against NRRL 1951 three main categories, labeled by different colors, including 9 second level categories are statistically overrepresented. **b** *A. chrysogenum*: For the comparison of A3/2 against ATCC 11550, five main categories including eight second level categories are statistically enriched (adjusted *p-*value < 0.05). *White* and *grey* bars indicate up and downregulated genes respectively. Dashed lines indicate not significantly enriched categories

Within the main category ‘metabolism,’ several second level categories where enriched in the data sets from both fungi. They share enrichment of the three categories ‘amino acid metabolism,’ ‘C-compound and carbohydrate metabolism’ and ‘secondary metabolism’ (Fig. 3a and b). In *A. chrysogenum*, genes from the latter two categories are significantly present in the set of up- and downregulated genes. In *P. chrysogenum,* however, ‘C-compound and carbohydrate metabolism’ is up- and ‘secondary metabolism’ is downregulated. Furthermore, in *P. chrysogenum*, the second level categories ‘nitrogen, sulfur and selenium metabolism’ and ‘lipid, fatty acid and isoprenoid metabolism’ are solely enriched within the upregulated genes.

Changes in amino acid metabolism might play an important role in the supply of energy and building blocks for β-lactam production. The pathways of branched-chain amino acid degradation are partly upregulated in both production strains. The first step of these pathways is catalyzed by branched-chain amino acid aminotransferases. Gene expression of *EN45_095580* and *ACRE_072730* encoding a branched-chain amino acid aminotransferase are 73.5-fold and 3.5-fold upregulated in the corresponding production strains, respectively. Mitochondrial branched-chain amino acid degradation supplies succinyl-CoA for the tricarboxylic acid (TCA) cycle. A branched-chain alpha-keto acid dehydrogenase complex, consisting of three subunits (E1-E3) catalyzes the last step of this degradation. Two of these subunits, E1 encoded by *EN45_092650* (4-fold) and *ACRE_031080* (6.1-fold) and the E2 encoded by *EN45_100120* (8.6-fold) and *ACRE_042000* (2.8-fold), are transcriptionally upregulated in the corresponding production strains. Furthermore, *EN45_082340* and *ACRE_040000* encoding predicted 3-hydroxyisobutyrate dehydrogenases catalyzing the conversion of 3-hydroxy-2-methylpropanoate to 2-methyl-3-oxopropanoate within L-valine degradation were also 9.8- and 2.6-fold upregulated, respectively. This might be because all branched-chain amino acids share common biosynthetic steps, but β-lactam production is only dependent on L-valine biosynthesis. All excess amino acid products not required for β-lactam biosynthesis might be used for energy metabolism driving energy-intensive β-lactam biosynthesis. Differential regulation of a branched-chain amino acid aminotransferase was also recently shown for the production strain *A. chrysogenum* 84–3–81–41 [11]. In this fungus, L-valine biosynthesis comprises four steps, some of which are differentially expressed in the industrial strain. In A3/2 however, we found that the acetolactate synthase (*ACRE_040710,* 3.0-fold) and a dihydroxy-acid dehydratase (*ACRE_066750,* 7.0-fold) genes are differentially regulated, both of which are responsible for the first or third catalytic step of L-valine biosynthesis. These genes are not differentially expressed in P2niaD18, but we found differential expression for genes involved in L-lysine synthesis. Fungi, in contrast to bacteria and plants, use the α-aminoadipic acid pathway for *de novo* L-lysine biosynthesis. Within this pathway, α-aminoadipic acid is the name-giving key metabolite that marks the branch point between primary and secondary metabolism. The α-aminoadipate reductase, using α-aminoadipic acid as substrate for L-lysine synthesis, is encoded by *EN45_099160* (*lys2*). This gene is expressed 4.6-fold higher in the P2niaD18 strain. A second gene, EN45_099570 (lys7), coding for a saccharopine reductase, which catalyzes the subsequent step in L-lysine synthesis, is expressed 3.7-fold higher. This stands in contrast to previous reports wherein production strains showed decreased activity of α-aminoadipate reductase, and deletion of *lys2* led to penicillin overproduction [49, 50]. However, activation of these steps under depleted conditions might supply building blocks for β-lactam synthesis. It was shown that these steps are reversible and strains unable to synthesize α-aminoadipic acid utilize L-lysine to synthesize α-aminoadipic acid for penicillin biosynthesis [51].

In conclusion, genes involved in amino acid metabolism are similarly regulated in both production strains, while others seem to have species-specific expression changes. This reflects the selective adaptation of two distantly-related organisms towards β-lactam production. To optimize L-valine biosynthesis in both fungi, we suggest inactivating competing pathways. L-valine is synthesized via four enzymatic reactions from two pyruvate molecules. Beside L-valine formation, these enzymes also catalyze the biosynthesis of L-isoleucine from pyruvate and ketobutyrate. Furthermore, ketoisovalerate, the last intermediate in L-valine biosynthesis, is used for L-leucine and D-pantothenate formation. This situation offers several options to optimize L-valine production by inactivation of competing pathways. In prokaryotic *Corynebacterium glutamicum*, inactivation of L-isoleucine biosynthesis by deletion of *ilvA*, which is responsible for synthesis of ketobutyrate, leads to increased L-valine formation. Moreover, inactivation of D-pantothenate biosynthesis in the same background results in even further enhanced L-valine production under D-pantothenate-limiting conditions [52]. The use of weak promoters instead of gene deletion avoids the construction of auxotrophic strains that are dependent on amino acid supplementation [53]. Reduced biosynthesis of other branched-chain amino acids is advantageous for producer strains in two alternative respects. On the one hand, metabolic flux is shifted towards L-valine biosynthesis resulting in a higher availability of L-valine; on the other hand, lower production of by-products reduces energy consumption for recycling pathways.

**Fig. 4:**
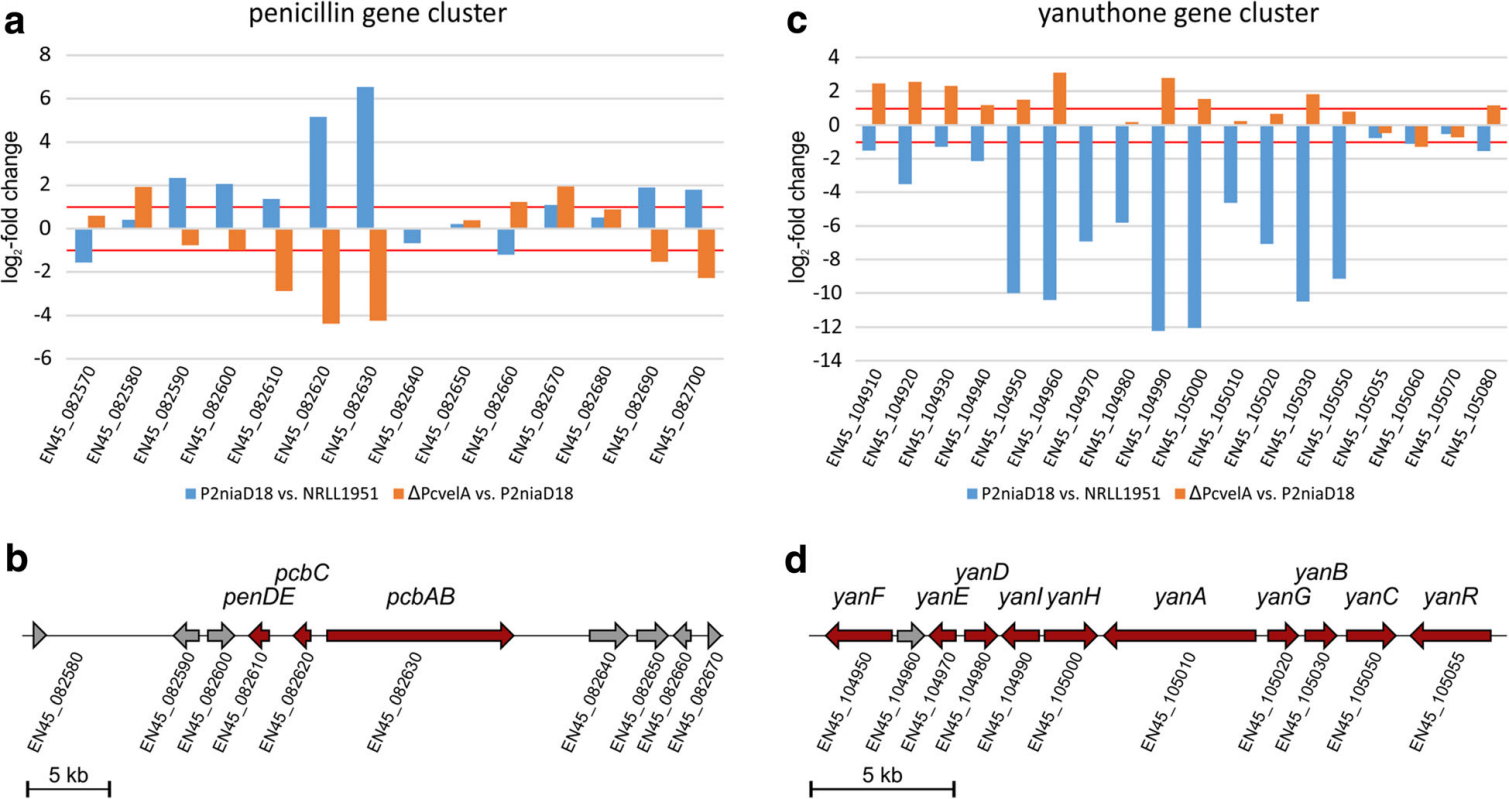
Changes in gene expression of secondary metabolite gene clusters in *P. chrysogenum*. Differential gene expression in *P. chrysogenum* was examined by pairwise comparison of the production strain with either wild-type or velvet deletion strain. As significant examples the gene cluster relevant for penicillin and yanuthone synthesis are shown. **a** Expressional changes and **b** cluster organization of the penicillin biosynthesis gene cluster and adjacent genes. **c** Expressional changes and **d** cluster organization of the yanuthone biosynthesis gene cluster. *Blue* bars represent the expressional change (log_2_-fold) comparing the industrial P2niaD18 with the wild type NRRL 1951, while *orange* bars represent the expressional changes induced by the velvet gene deletion. Relevant biosynthetic genes are indicated in *red*

**Fig. 5:**
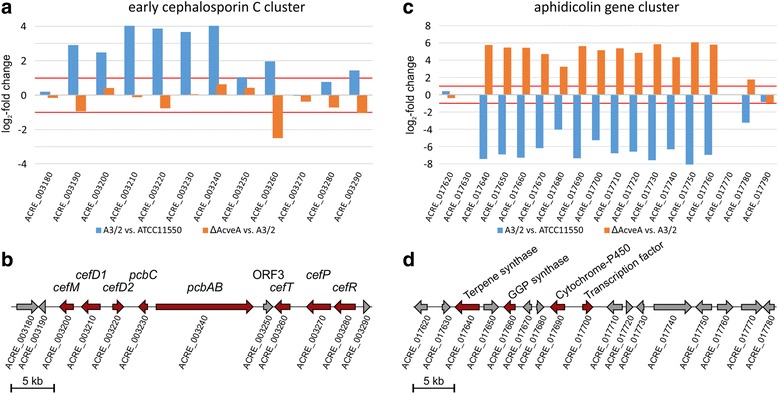
Changes in gene expression of secondary metabolite gene clusters in *A. chrysogenum*. Differential gene expression in *A. chrysogenum* was examined by pairwise comparison of the production strain with either wild-type or velvet deletion strain. The early cephalosporin C cluster as well as the aphidicolin cluster are given as representative examples. **a** The expressional changes of the early cephalosporin C cluster and **b** its genomic organization are given. **c** Expressional changes of the aphidicolin biosynthesis genes and **d** organization of cluster associated and adjacent genes. *Blue* bars represent the expressional change (log_2_ fold) comparing the industrial strain A3/2 with the wild type ATCC 11550, while *orange* bars represent the expressional changes induced by the velvet gene deletion. Genes relevant for biosynthesis are indicated in *red*

We have further analyzed additional second level categories such as ‘secondary metabolism.’ In *P. chrysogenum,* this category is present only in the set of downregulated genes, while in *A. chrysogenum*, both up- and downregulated genes are enriched. During functional annotation, 42 downregulated genes from *P. chrysogenum* as well as 114 upregulated and 138 downregulated genes from *A. chrysogenum* were assigned to this category. Since many secondary metabolite genes are organized in clusters, we looked for the corresponding clusters in *P. chrysogenum* and used existing data from previous work with *A. chrysogenum* [54]. In *P. chrysogenum,* 27 of the 54 secondary metabolite clusters are differentially regulated in the industrial strain. Similarly, in *A. chrysogenum* 23 of 41 secondary metabolite clusters are differentially regulated. As expected, clusters directly associated with β-lactam production are upregulated in both fungi. The penicillin gene cluster from *P. chrysogenum* (*EN45_082610* – *EN45_082630* and *EN45_082990* – *EN45_083010*), which is duplicated in the investigated production strain, is more than four-fold upregulated (Fig. 4a and b), while the single copy of the early cephalosporin C cluster from *A. chrysogenum* (*ACRE_003200* – *ACRE_003290*) is nearly sixteen-fold upregulated (Fig. 5a and b). These results were further verified by a RT-qPCR analysis (Additional file 3: Figure S2). In addition to genes in the early cephalosporin C cluster, another gene (*ACRE_003190*) upstream of *cefM* shows corresponding transcriptional changes during strain improvement. Therefore, we hypothesize that this gene, which encodes an uncharacterized protein with predicted N-acetyltransferase activity, belongs to the early cephalosporin C cluster. Aside from the well-characterized β-lactam biosynthesis genes, additional genes involved in the degradation of desired products are major factors minimizing production yield in both fungi. In *A. chrysogenum,* cephalosporin C acetylhydrolases can degrade cephalosporin C to the commercially irrelevant side-product deacetylcephalosporin C [55]. Genes encoding proteins involved in this process (*ACRE_083500*, *ACRE_083460*, *ACRE_049960*) are 4.9- to 1176.3-fold downregulated in A3/2. In *P. chrysogenum*, the orthologous gene (*EN45_076310*), which was characterized as a major allergen from *P. notatum*, is also 7.0-fold downregulated [56]. The accumulation of deacetylcephalosporin C is a serious problem for downstream processes since it cannot be separated from the target metabolite cephalosporin C [55]. We propose that this is also true for penicillin purification from *P. chrysogenum* culture supernatants.

Other secondary metabolite clusters were downregulated close to the point of no expression in both industrial organisms. The cluster with the largest expressional decrease in P2niaD18 is a polyketide synthase cluster (*EN45_104960* – *EN45_105055*) that shows strong similarity to the recently described yanuthone biosynthesis gene cluster from *Aspergillus niger* [57] (Fig. 4c and d). Ten genes constitute the core of this cluster, nine of which are strongly downregulated in the industrial strain, including the core gene encoding a polyketide synthase (*EN45_105010*). The read counts for the two cytochromes P-450 (*EN45_105000* and *EN45_105050*), as well as for the decarboxylase (*EN45_105030*) and the O-mevalon transferase (*EN45_104990*) were close to the point of no expression. In A3/2, a gene cluster (*ACRE_017620* - *ACRE_017900*) with similarity to the aphidicolin cluster from the pathogenic fungus *Phoma betae* was significantly downregulated [58] (Fig. 5c and d). Read counts for core genes of this cluster, were also reduced to almost zero. These are the genes for a terpene synthase (*ACRE_017640*), a geranylgeranyl diphosphate synthase (*ACRE_017660*), a cytochrome P-450 (*ACRE_017690*) and the cluster regulating transcription factor (*ACRE_017700*). This rather restricted expression of only a few metabolite clusters may result in the optimized use of energy and precursors for the efficient production of selected compounds such as β-lactams. Therefore, metabolic flux seems to be reorganized in production strains. Our assumption is supported by a recent application of the RAVEN (Reconstruction, Analysis and Visualization of Metabolic Networks) Toolbox, which was used to reconstruct a genome-scale metabolic model for *P. chrysogenum* (Wisconsin54-1255) [59]. In this application, gene expression data were incorporated into a map to illustrate the correlation between flux and gene expression. The authors found a total of 58 fluxes to be significantly changed between high and low production strains, and 612 genes were differentially expressed. Out of those, 36 reactions were identified as having significantly higher flux and up-regulated genes. This result implicated that these reactions are likely to have transcriptional regulation of their fluxes.

### Expression of cellular transport-associated genes is downregulated in industrial strains

Within the first level category, ‘cellular transport,’ genes related to the second level category ‘transport facilities,’ are enriched in both production strains. Further, the second level category, ‘transported compounds (substrates),’ is solely enriched in *P. chrysogenum*. All of these categories are only significantly enriched in the set of downregulated genes.

Aside from transport-related genes, which are regulated only in one or the other of the two organisms, we were able to identify a group of orthologous genes encoding transporters. All of them were downregulated during strain improvement with different levels of regulation ranging from 2.8- to 630.3-fold decreased expression. The strongest downregulated gene pair (*EN45_068550*, *ACRE_048130*) encodes a predicted Major-Facilitator-Superfamily (MFS) multidrug transporter and is located within orthologous polyketide synthase gene clusters involved in sorbicillinoid biosynthesis, which constitute yellow pigments found in wild-type strains of both organisms, as well as in *Trichoderma reesei* [60, 61]. The transcript level of this transporter gene was close to zero in both, *P. chrysogenum* and *A. chrysogenum*. Other backbone genes were also downregulated in *A. chrysogenum*, while expression of the corresponding core genes is unaltered in *P. chrysogenum*.

Sorbicillinoid biosynthesis was eliminated in both fungi to improve downstream processing during strain improvement programs. Interestingly, alterations between production strains of different lineages are diverse. While derivatives obtained from Wisconsin 54–1255 show point mutations within structural genes of the cluster, P2niaD18 carries a wild-type copy of this cluster [15, 20]. Our RNA-seq data led us to conclude that pigment production is transcriptionally repressed. Similarly, *A. chrysogenum* A3/2 carries a wild-type version of the corresponding cluster, which is severely downregulated as compared to the wild-type strain ATCC 11550.

Aside from the aforementioned transporter genes, others are significantly downregulated in both β-lactam producer strains. For example, we found two putative ATP-binding-cassette (ABC)-2 type transporters (*EN45_056900*, 90.5-fold; *ACRE_038160*, 2.8-fold; *EN45_036600*, 26.0-fold; *ACRE_037640*, 8.6-fold). Orthologs from these two transporters in *A. oryzae* (*AO090010000219*, orthologous to *EN45_056900*) and *Aspergillus fumigatus* (*abcC*, orthologous to *EN45_036600*) are upregulated under exposure to benomyl (*A. oryzae*) and azole, itraconazole, voriconazole, and posaconazole (*A. fumigatus*) [62–65]. We further identified a MFS multidrug transporter (*EN45_080850*, 42.2-fold; *ACRE_043540*, 3.5-fold), which is upregulated in *A. nidulans* and *A. oryzae* under hypoxic growth conditions [66] and a vacuolar iron transporter (*EN45_018160*, 16-fold; *ACRE_060860*, 4.3-fold) that mediates vacuolar iron storage in *Saccharomyces cerevisiae* (CCC1) and is important for metal and toxaphene tolerance [67]. All these transporters have functions that are important for stress tolerance. We hypothesize that these are dispensable under the constant growth conditions, which occur for example during industrial fermentations. The supply of a reduced number of transporters conserves energy, which can be used for β-lactam antibiotic production. Another homologous pair of transporter genes (*EN45_112050*, 36.8-fold; *ACRE_070310*, 32.0-fold) is downregulated in both production strains. An orthologue in *Candida albicans* (HGT7) is described as a high affinity glucose transporter [68]. It is known that glucose is able to inhibit β-lactam antibiotic production in both fungi and thus downregulation might lead to a reduced glucose uptake, which can be advantageous for improved production [69, 70]. In addition to transcriptional regulation of transporter-encoding genes, other factors are known to modulate the transport activity of a cell. For example, arrestin-related trafficking adaptors can ubiquitinate transport proteins in response to extrinsic stimuli, which leads to turnover of the marked transporters by endocytosis. *EN45_103660* and *ACRE_058500* encode a homolog of the arrestin-related trafficking adaptor ArtG from *A. nidulans* and are 9.8 and 5.3-fold overexpressed during the strain improvement process, respectively [71].

In summary, transport appears to be an important aspect within strain improvement processes of both fungi, which share a set of differentially regulated transport-related genes. As may be expected, other transporter genes are differentially expressed only in one or the other investigated organisms. These results seem to be the consequence of a selective uptake of substrates and reduced efflux of usable compounds to conserve energy.

### The expression of genes involved in cell rescue, defense and virulence is downregulated in industrial strains

The third enriched first level category in both production strains is ‘cell rescue, defense and virulence.’ Within this first level category, second level categories ‘disease, virulence and defense’ and ‘detoxification’ are enriched. These categories are only significantly enriched in the set of downregulated genes for *P. chrysogenum*, while in *A. chrysogenum,* both second level categories are enriched in sets of down- and upregulated genes. Within these second level categories, we identified different groups of similar genes. These are genes for transport-related proteins like ABC-transporters and MFS-transporters as well as proteins related to secondary metabolism like cytochrome P-450, non-ribosomal peptide synthetases and polyketide synthases. Many of these genes that belong to ‘disease, virulence and defense’ and ‘detoxification’ categories can also be assigned to other functional categories, such as ‘secondary metabolism’ or ‘transport facilities.’ The latter two categories are significantly enriched in both industrial strains, as was described in the previous section. Examples, of assignment to more than one category are the orthologues *EN45_036600* and *ACRE_037640*, which are strongly downregulated during strain improvement in both organisms (*EN45_036600*, 25.5-fold; *ACRE_037640*, 8.7-fold). The corresponding second level categories are ‘transport facilities’, ‘secondary metabolism,’ ‘disease, virulence and defense,’ and ‘detoxification.’ Due to diverse mechanisms that are involved in cell rescue processes, it is unsurprising that related functional categories are enriched within our data set. Our data are consistent with recent results of a comparative proteome analysis that used NRRL 1951 and the moderate penicillin producer Wisconsin 54–1255 as sources [72, 73]. Although the benefit of decreased expression of these genes has not been unequivocally clarified, it can be hypothesized that decreased expression of genes related to cell rescuing processes is due to optimized energy consumption. Recent studies in the bacterium *Streptomyces avermitilis* demonstrated that disruption of genes involved in the osmotic stress response led to increased antibiotic production [74]. We suggest that downregulation of genes within these categories is a result of adaption to constant growth conditions that are typically used during industrial strain selection programs and fermentations, and that changes in cell rescue processes, like the stress response and detoxification, might contribute to improved β-lactam production in industrial strains.

### Strains from improvement programs show major genome rearrangements

We identified an 88 kb spreading region (scaffold 111) in the industrial *A. chrysogenum* strain that appeared not to be transcribed, since none of the reads mapped to scaffold 111. This led us to conclude that this genomic region, which is transcriptionally expressed in the wild-type strain, is absent in the production strain. To test this hypothesis, we performed PCR amplification of regions across scaffold 111 (Additional file 4: Figure S3). None of the selected oligonucleotide primers generated an amplicon with genomic DNA from the production strain, while wild-type DNA delivered the expected fragment. Thus, scaffold 111, carrying 29 protein-coding genes, is absent in A3/2. Among the lacking genes are those for transporters, kinases, a glutathione S-transferase, a zinc finger transcription factor and a histidine sensor kinase. The latter, encoded by *ACRE_073970*, is 72% identical to Os-1, a sensor histidine kinase from *N. crassa,* which is important for the osmotic stress response. While *N. crassa* only has one gene encoding Os-1, *A. chrysogenum* carries a paralog encoding a protein with 80% identity to Os-1. Therefore, loss of *ACRE_073970* likely does not lead to a complete loss of osmotic sensing, but might reduce sensing capacity. As mentioned above, a reduced stress response may save energy under controlled fermentation conditions and therefore lead to improved production of secondary metabolites. We previously showed via pulsed-field gel electrophoresis analysis that strains with different cephalosporin C production rates have undergone major genomic reorganizations [75]. This is reminiscent of genome rearrangements that were recently demonstrated for *P. chrysogenum* P2niaD18 [20, 76] and other industrial penicillin producer strains [77, 78]. These major chromosome rearrangements are most likely the result of extensive random mutagenesis that occurred during repeated strain improvement programs.

### In both fungi, a large set of Velvet-regulated genes are differentially expressed during strain improvement

Velvet is a global regulator of secondary metabolism in filamentous fungi. Velvet was first discovered in *A. nidulans* as an inhibitor of light-dependent conidiation [79], and was later shown to control the biosynthesis of a large number of metabolites in diverse filamentous ascomycetes [80]. In recent years, genetic, biochemical and molecular work has demonstrated that Velvet interacts with methyltransferases and other velvet domain-containing proteins in a multi-subunit complex [29, 80]. Velvet is characterized by a 150 amino acid protein domain, which so far has only been detected and characterized in filamentous fungi. It was recently shown that this domain is essential for DNA binding and is necessary for protein dimerization within the Velvet complex [81, 82]. Because of Velvet’s broad regulatory spectrum and its impact on secondary metabolism, we sought to analyze the regulatory role of Velvet in both industrial fungi. A particular focus was to determine Velvet regulated genes that were also differentially expressed after strain improvement.

**Fig. 6:**
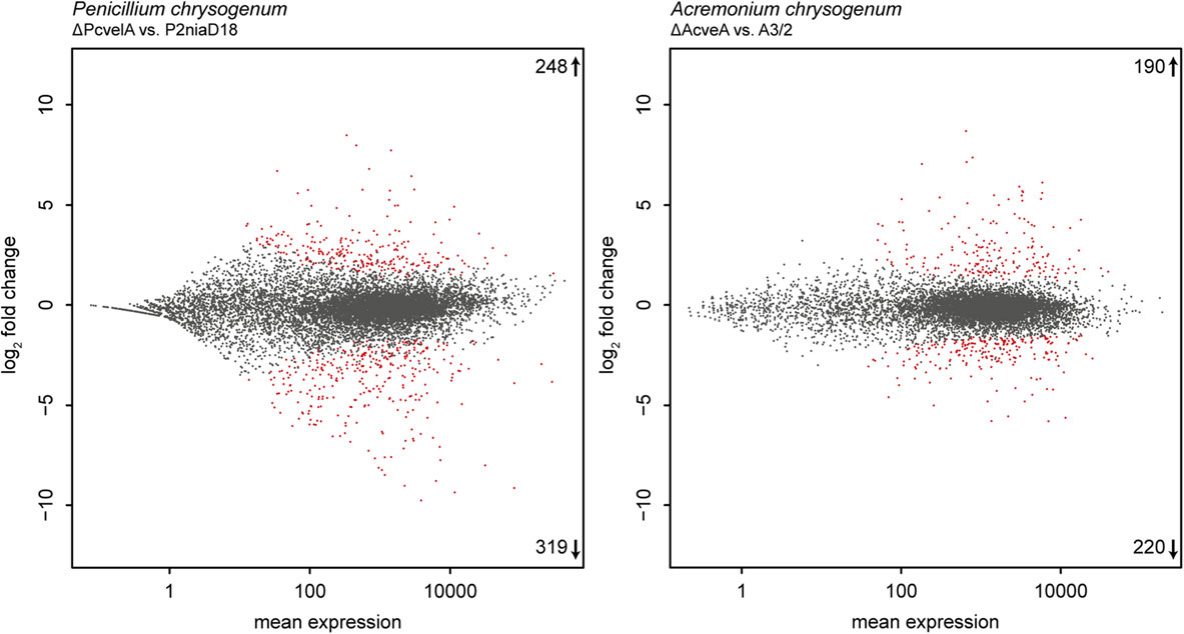
Differential gene expression in *velvet* deletion strains. MA-plots for gene expression data from comparison of velvet deletion strains from *P. chrysogenum* and *A. chrysogenum* compared to the corresponding industrial production strain. Log_2_-fold change as well as the mean expression out of two replicates are shown. *Red* dots represent statistical significant differential expressed genes, while grey dots mark not differentially expressed genes

**Fig. 7:**
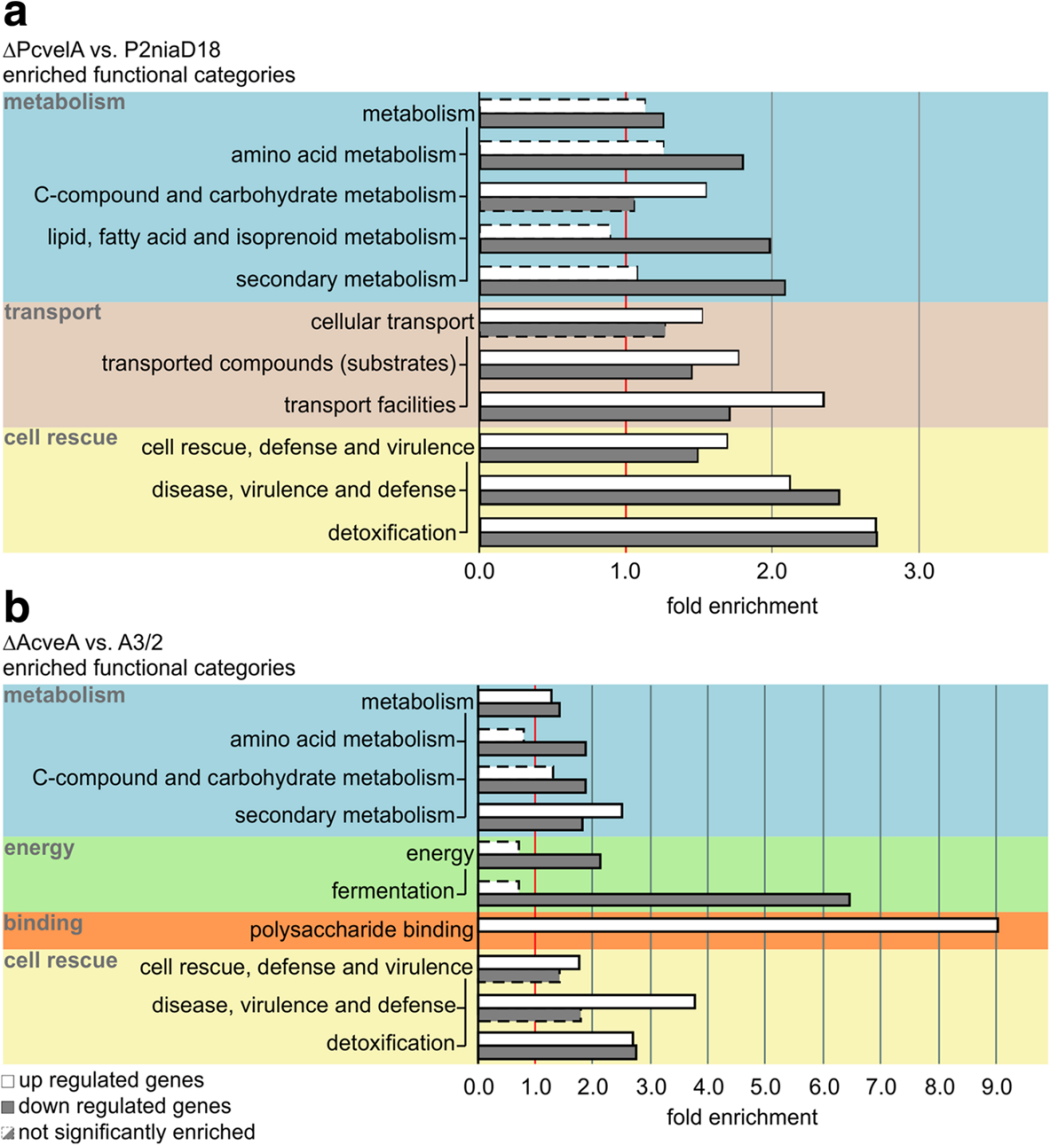
FungiFun2 enrichment analysis and functional categorization (FunCat) of differentially expressed genes in velvet deletion strains. Deletion strains were compared with the corresponding industrial strains. **a** *P. chrysogenum*: Within the comparative data set of ΔPcvelA against P2niaD18 three main categories, labeled by different colors, including eight second level categories are statistically overrepresented. **b** *A. chrysogenum*: For the comparison of ΔAcveA against A3/2, four main categories including seven second level categories are statistically enriched (adjusted *p-*value < 0.05). White and grey bars indicate up and downregulated genes respectively. Dashed lines indicate not significantly enriched categories

To elucidate Velvet-dependent transcriptional changes, RNA was isolated from *P. chrysogenum* and *A. chrysogenum* strains lacking the *velvet* gene (*PcvelA*, *AcveA*) [30, 32]. Subsequent high throughput RNA-sequencing detected 567 differentially regulated genes in ΔPcvelA, which constitutes 5% of the annotated genes. In ΔAcveA, 410 differentially regulated genes were detected, which constitutes 4.4% of the annotated genes. A total of 248 (43.7%) of the differentially regulated genes from *P. chrysogenum* are upregulated and 319 (56.3%) are downregulated, while in *A. chrysogenum,* 190 (46.3%) are upregulated and 220 (53.7%) are downregulated (Fig. 6). Differentially expressed genes were assigned to functional categories based on the FunCat database and used for subsequent enrichment analysis. In the ΔPcvelA strain, five second level FunCat categories are significantly enriched in the set of overexpressed genes, while seven second level categories were overrepresented in the downregulated gene set (Fig. 7a). Enrichment analysis of gene sets from the ΔAcveA strain showed enrichment of four second level FunCat categories in the upregulated set and five second level categories in the downregulated set (Fig. 7b). Both organisms share enrichment of the second level categories ‘disease, virulence and defense’ and ‘detoxification’ in the set of upregulated genes and overrepresentation of second level categories ‘secondary metabolism,’ ‘detoxification,’ and ‘amino acid metabolism’ in the downregulated gene set. In addition to the shared enriched categories, *P. chrysogenum* shows an overrepresentation of the categories ‘C-compound and carbohydrate metabolism,’ ‘transported compounds (substrates)’ and ‘transport facilities’ within the set of upregulated genes and an enrichment of ‘lipid, fatty acid and isoprenoid metabolism,’ ‘transported compounds (substrates),’ ‘transport facilities’ and ‘disease, virulence and defense’ in the set of downregulated genes. The second level FunCat category ‘polysaccharide binding’ is solely enriched within the group of upregulated genes, and the categories ‘fermentation’ and ‘C-compound and carbohydrate metabolism’ are overrepresented exclusively in the set of downregulated genes in ΔAcveA.

Thus, Velvet regulates common functional categories, like secondary or amino acid metabolism in the two fungi investigated herein. However, some functional categories like transport or energy-related categories are only enriched in one fungus for example, energy in *A. chrysogenum* or transport in *P. chrysogenum*.

### Velvet has a major impact on the expression of approximately 50% of all secondary metabolite clusters

**Fig. 8:**
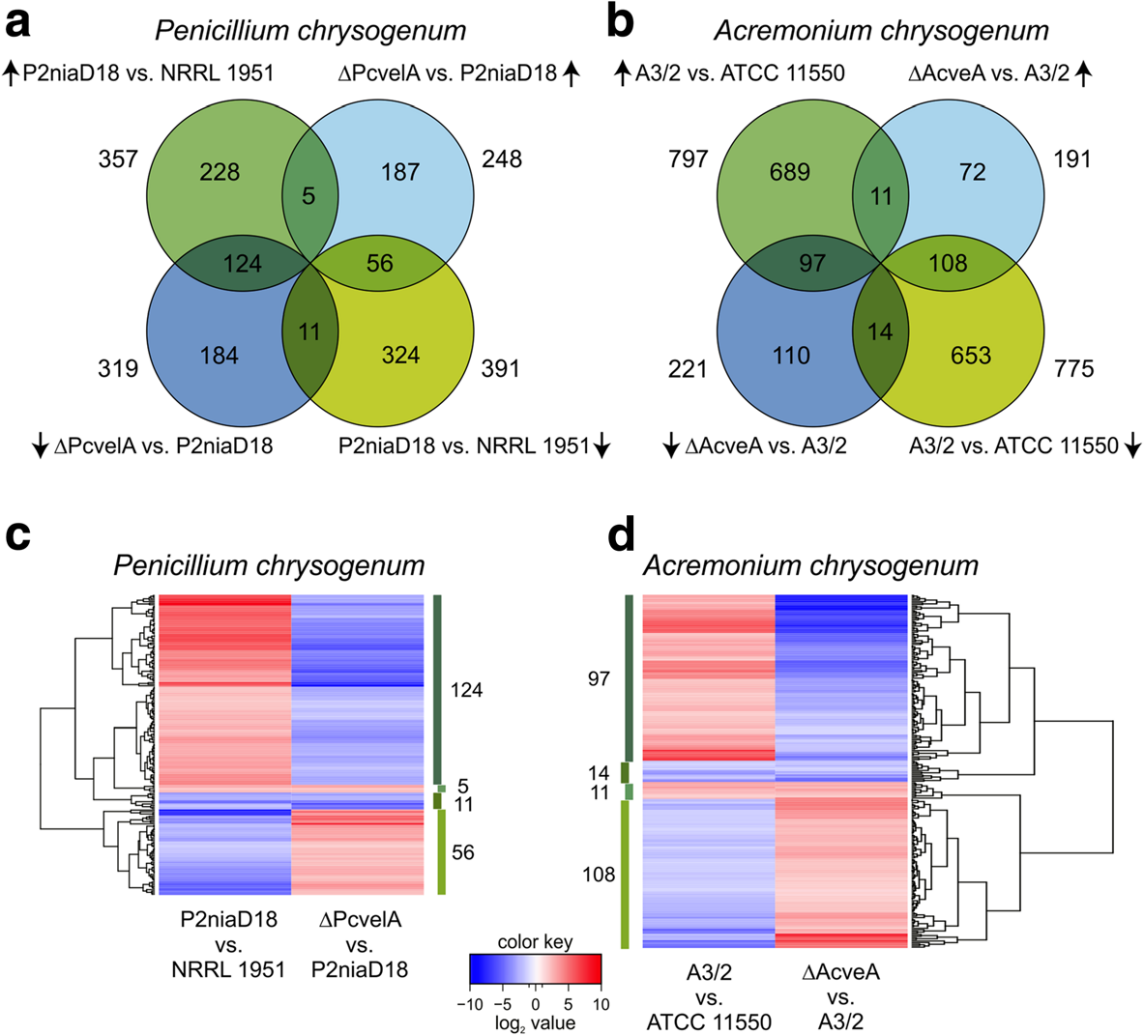
Differential gene expression in industrial strains compared to their corresponding wild-type and velvet deletion strains. **a** and **b** Venn-diagrams showing intersection of differentially expressed genes from strains as indicated. Arrows mark direction of the regulatory effects. **c** and **d** Hierarchical cluster analysis of all differential expressed genes within the intersections as indicated. Intensity of *blue* and *red* colors illustrate the degree of differential gene regulation

Secondary metabolism provides advantages for a given microbe in defined ecological niches. However, the production of these metabolites is energy consuming and therefore must be strictly regulated. In industrial production processes, microorganisms should invest most of their energy to obtain optimal yield of the desired secondary metabolite. During strain improvement, both fungi underwent expressional changes of secondary metabolite clusters. From the analysis of *velvet* deletion strains, we conclude that Velvet controls the expression of about 50% of all secondary metabolite clusters in both fungi. In *P. chrysogenum* roughly half of differentially expressed clusters are up- or downregulated, which indicates that Velvet functions as both a positive and a negative regulator. In contrast, 89% of the differentially expressed clusters in *A. chrysogenum* are upregulated in the *velvet* deletion strain*,* suggesting that Velvet acts mainly as a negative regulator of secondary metabolism. Our comparative expression analyses in Figs. 3, 7 and 8 show that strain improvement programs largely alter the expression of genes that are targets of Velvet. We propose that down regulation of clusters not linked to β-lactam production might result in more efficient energy and precursor consumption, thus leading to improved β-lactam synthesis. In *P. chrysogenum,* upregulation of single genes from a metabolite cluster may increase product formation through optimized transport, catalytic activity, or supply of intermediates. Our results assign Velvet an important role in secondary metabolism, which is consistent with its regulatory function in other filamentous fungi [80, 83].

### Velvet target genes are differentially expressed in improved β-lactam production strains

Via pair-wise comparison, we identified a large set of differentially expressed genes that belong to diverse functional categories (Figs. 3 and 7). We determined the intersection of genes that are altered in both, production strains and *velvet* mutants. Figure 8 shows the number of genes found in the intersection and expression changes in both organisms. For *P. chrysogenum*, we found 196 genes that are regulated in both data sets. This corresponds to 26.2% of 748 differentially expressed genes found during strain improvement. From this intersection, 92% of all transcripts are inversely regulated in both data sets. In *A. chrysogenum*, this overlap consists of 230 genes, which makes up 14.6% of differentially expressed genes found during strain improvement. A comparison of expressional changes within this intersection showed a rather high proportion (89%) that are inversely regulated in the two data sets, which corresponds to our findings in *P. chrysogenum*. Examples of these expressional patterns are secondary metabolite clusters involved in penicillin (Fig. 4a), yanuthone (Fig. 4c) and aphidicolin (Fig. 5c) production. Other cellular processes like ‘amino acid metabolism’ (both fungi), ‘lipid, fatty acid and isoprenoid metabolism’ (*P. chrysogenum*) and ‘fermentation’ (*A. chrysogenum*) are deregulated in the same manner. Thus, in both species, strain improvement and Velvet affect the expression of a large set of genes in a similar manner. This assigns Velvet an important role during strain improvement and supports the hypothesis that regulatory changes rather than changes in the nucleotide sequences of structural genes has led to improved product titers. We would like to emphasize that both production strains carry a wild-type version of the velvet gene, lacking any point mutation and significant transcriptional alterations. In this case, a complete deletion of the velvet gene, resulted in a drastic reduction of the antibiotic titer [30, 32]. This is different to a previous report on another *P. chrysogenum* producer strain (DS17690), showing that strain improvement programs have targeted the velvet gene, and thus leading to a truncated version of Velvet [10]. A subsequent complete deletion of the velvet gene in DS17690 background did not result in a major reduction of the penicillin titer [84].

### Varying composition of the Velvet complex

As mentioned above, velvet forms various sub-complexes with other proteins that are responsible for different developmental programs, including primary and secondary metabolism or sexual and asexual development [34]. The composition of proteins is well studied in *P. chrysogenum* and other filamentous fungi such as *A. nidulans* and *Fusarium oxysporum* [29, 80, 85]*. P. chrysogenum* and *A. nidulans* belong to class Eurotiomycetes and share a homologous set of Velvet proteins. In *P. chrysogenum,* the Velvet complex consists of at least five proteins, namely PcVelA, PcVelB, PcVelC, PcVosA and the methyltransferase PcLaeA. All proteins show an interaction with PcVelA at given time points and therefore appear to be part of the Velvet complex [29]. We sought to determine whether the full set of Velvet homologous proteins is also encoded in the genome of the Sordariomycete *A. chrysogenum*. Aside from *AcveA*, we detected *AcveB* in *A. chrysogenum*. However, other homologous genes, such as *velC* and *vosA* or other genes encoding proteins with a velvet domain were not detected. This finding is consistent with reduced or altered Velvet complex in the closely related genus *Fusarium*, which lacks a VosA homologue. This reduced composition of the Velvet complex in some fungal species led us to speculate that other proteins have replaced the regulatory functions of conserved subunits of the Velvet complex.

## Conclusions

This study is a comparative gene expression analysis of two distantly related ascomycetous fungi, both of which are major industrial β-lactam antibiotic production strains. Here, we provide comparative transcript analyses of industrial strains that were pairwise compared to wild type or deletion mutants lacking the gene for *velvet*, a global transcriptional regulator. The analysis of twelve individual RNA-seq data sets showed that differential gene expression occurred in distinct functional categories in both industrial strains. Furthermore, we found that Velvet regulates genes assigned to these categories. Our data will be beneficial for strategies to identify new targets for concerted strain improvement programs.

Current strategies to enhance microbial metabolite production can be generally classified into four different strategies [86]. The first strategy is to enhance the expression of genes involved in biosynthesis of the target metabolite. This approach is demonstrated in both fungi by the several-fold upregulation of genes within the penicillin or early cephalosporin C clusters. We also show that expression of genes involved in L-valine and L-lysine metabolism were altered in *P. chrysogenum* and *A. chrysogenum*. While L-valine is a precursor for ACV synthetase, L-lysine synthesis depends on the conversion of α-aminoadipic acid. Therefore, we expect that altered expression of genes involved in metabolism of both amino acids are directly or indirectly related to increased β-lactam production.

The second strategy is to repress or to eliminate reactions that degrade or convert essential precursors or target metabolites. Several acetylhydrolases (*ACRE_083500*, *ACRE_083460*, *ACRE_049960*) that can degrade cephalosporin C to deacetylcephalosporin C are drastically downregulated in the industrial strain. We propose that orthologous genes in *P. chrysogenum*, like *EN45_076310*, might also be involved in penicillin degradation. Further investigation in this context might be highly relevant for fermentation processes.

The third strategy is to repress the expression of genes from other secondary metabolite pathways, like yanuthone in *P. chrysogenum* or aphidicolin in *A. chrysogenum*. This might contribute to increased energy flux towards β-lactam biosynthesis. The elimination of competitive pathways and a redistribution of chemical compounds, induced by altered expression of genes involved in cellular transport processes might have contributed positively to increased β-lactam production in both industrial strains.

The fourth strategy is to optimize transport processes to increase the uptake of precursors and remove target metabolite or interfering products. Products like deacetylcephalosporin C or sorbicillinoids, yellow pigments present in wild-type strains, interfere with purification of the target products. As discussed above, sorbicillinoid biosynthesis is absent in both industrial strains and might be the result of downregulated cluster-specific transporters.

Our data will support the above mentioned strategies to optimize metabolite production of diverse microbial production strains [86, 87].

## Methods

### Strains and culture conditions

All *P. chrysogenum* and *A. chrysogenum* strains used in this study are listed in Table 1. For expression analysis, the strains were grown as independent biological duplicates at 27 °C and 120 (*P. chrysogenum*) or 180 (*A. chrysogenum*) rpm for 3 days in complete culture medium (CCM) as described previously [28]. Inoculations were performed with 1 × 10^7^ colony forming units for *P. chrysogenum* and 200 mg harvested mycelium for *A. chrysogenum*.

**Table 1:** Fungal strains and mutants used in this investigation

Strains	Relevant genotypes	Source
NRRL 1951	Wild type	[99]
P2niaD18	Production strain, *niaD* ^−^	[100]
ΔPcvelA	Δ*PcvelA*::*ble* Δ*Pcku70*::*nat1 niaD* ^−^	[101]
ATCC 11550	Wild type	[102]
A3/2	Production strain	[103]
ΔAcveA	Δ*veA*::*hygB*	[32]

### RNA isolation and sequencing

*P. chrysogenum* and *A. chrysogenum* mycelia were harvested and isolation of total RNA was performed as described recently [32]. RNA from biological duplicates of each strain was delivered for custom sequencing. Strand specific library preparation and single end sequencing (read length 50 nt) was performed on the Illumina HiSeq 2500 platform by GATC Biotech (Konstanz, Germany).

### Data processing

Quality of raw reads were analyzed using FastQC report. Due to sufficient quality, reads were used for the alignment with TopHat v2.1.0 [88] to the respective genomes of *P. chrysogenum* (P2niaD18 [20]) and *A. chrysogenum* (ATCC 11550 [54]) without any quality trimming. A summary on read output and mapping rates are given in the supplementary material (Additional file 5: Table S2).

### Genome annotation used for expressional quantification

*A. chrysogenum* annotation, available at DDBJ/ENA/GenBank under the accession no. JPKY00000000.1, was used for expressional quantification.

For *P. chrysogenum*, *ab initio* gene prediction software Augustus v3.2.1 [89] and geneMark-ES v.4.32 [90] were used together with Braker1 [91] to generate a first RNA-seq based-annotation. The information from the Braker1 annotation as well as protein information from fungal proteins in the SwissProt database were merged using the Maker annotation pipeline v.2.31.8 [92]. This annotation was used for read counting and expression quantification. The annotation is deposited at DDBJ/ENA/GenBank under the accession no. JMSF00000000.1.

### Expression quantification

Read count matrices were generated strand specific using R [93] and summarizeOverlaps from the GenomicAlignments [94] package in union mode. Using the gplots 2.17.0 package [95], we performed a principal components analysis (PCA) from all our RNA-seq data sets (Additional file 6: Figures S4a and b) to confirm the reproducibility and expressional distance of our biological samples. Normalization and calculation of expressional changes was done using DEseq2 [42]. Genes were regarded as differentially expressed if expression change was more than two-fold and the adjusted p-value was below 0.1. Differential gene expression was verified by RT-qPCR for selected target genes (Additional file 3: Figure S2).

### Reverse-transcription quantitative polymerase chain reaction

Reverse-transcription quantitative polymerase chain reaction (RT-qPCR) was performed as described previously [96], oligonucleotides used as primers are given in Additional file 7: Table S3.

### Hierarchical clustering

Hierarchical clustering of differentially expressed and orthologous genes from *P. chrysogenum* and *A. chrysogenum*, which were identified based on reciprocal BLAST analysis, and construction of heatmaps were performed using R [93] and the gplots 2.17.0 package [95].

### Functional categorization of differentially expressed genes and enrichment analysis

The functional categories (FunCat) [48] assigned to differential expressed genes were used for enrichment analysis using FungiFun2 [47]. Enriched terms were searched for both organisms using hypergeometric distribution with FDR p-value filtering mode using a cutoff of 0.05. Indirectly annotated top categories were also considered for the analysis.

### Identification of secondary metabolite clusters

Genome-wide identification of secondary metabolite cluster was performed using antiSMASH v3.0 [97]. Based on the similar transcriptional level of clustered genes, the border of secondary metabolite clusters of interest were further defined.

### Phylogeny of PcbC

PcbC-like proteins were identified by tblastn analysis against fungal whole genome shotgun sequences (WGS) NCBI and blastp against non-redundant protein sequences based on the protein sequence of PcbC from *P. chrysogenum*. Derived protein sequences were used for phylogenetic analysis performed with MEGA7 [98]. With bootstrapping 1000 repetitions using the ‘neighbor-joining’ method.

## Additional files

Additional file 1: Figure S1. Alignment of the amio acid sequence of various PcbC orthologs. Predicted protein domains are indicated by colored boxes. Substrate and cofactor binding sites are marked by black or red squares. The premature stop codon in the pcbC ortholog from *P. ipomoeae* is indicated by a red asterisk. (TIF 3780 kb)

Additional file 2: Table S1. Summary of orthologous genes differentially regulated similarly in both production strains. Gene expression of similarly regulated orthologous genes grouped by their biological function; unique identifiers, fold changes, and functional descriptions are given. (XLSX 11 kb)

Additional file 3: Figure S2. Validation of RNA-seq data by RT-qPCRs. Expression levels of selected genes within the penicillin and yanuthone gene cluster from *P. chrysogenum* and within the early cephalosporin C and aphidicolin gene cluster from *A. chrysogenum* were confirmed by RT-qPCR. For each gene cluster, the change in expression (log_2_-fold) of three genes is shown in comparison to the results of the RNA-seq analyses. (TIF 612 kb)

Additional file 4: Figure S3. PCR amplification of regions across scaffold 111 from *A. chrysogenum*. The presence and absence of scaffold 111 within the genomic sequence of wild-type strain ATCC 11550 and industrial strain A3/2 was verified by PCR. Grey arrows indicate open reading frames. Black arrows mark primer binding sites for the three amplicons. (TIF 717 kb)

Additional file 5: Table S2. Summary of mRNA-seq read data. Raw sequencing output and number of aligned RNA-seq reads for biological replicates of *P. chrysogenum* and *A. chrysogenum* strains used in this work. (XLSX 10 kb)

Additional file 6: Figure S4. Principal component analysis (PCA) from all included RNA-seq data sets. (a) PCA-plot for all RNA-seq data sets from *P. chrysogenum*. (b) PCA-plot for all RNA-seq data sets from *A. chrysogenum*. (TIF 523 kb)

Additional file 7: Table S3. List of oligonucleotides. Oligonucleotides used for reverse-transcription quantitative polymerase chain reaction (RT-qPCR). Primer specificity and orientation are given (fw: forward; rv: reverse). (XLSX 10 kb)

## Abbreviations

ABC-transporter: ATP-binding-cassette transporter
ACV: δ-(L-α-aminoadipyl)-L-cysteinyl-D-valine
*AcveA*: Velvet gene from *Acremonium chrysogenum*
*AcveB*: Gene for Velvet subunit B from *A. chrysogenum*
ArtG: Arrestin-like protein G from *Aspergillus nidulans*
ATCC: American type culture collection
CCM: Complete culture medium
*cefD*: Gene for isopenicillin N epimerase
*cefE*: Gene for deacetoxycephalosporin C synthase
*cefEF*: Gene for deacetylcephalosporin C synthetase/hydroxylase
*cefF*: Gene for deacetoxycephalosporin C hydroxylase
FunCat: Functional catalogue
*ilvA*: Gene for L-threonine dehydratase
IPN: Isopenicillin N
*lys2*: Gene for L-aminoadipate-semialdehyde dehydrogenase
*lys7*: Gene for saccharopine reductase
MFS-transporter: major-facilitator-superfamily transporter
NCBI: National center for biotechnology information
NRRL: Northern regional research laboratory
Os-1: Histidine kinase from *Neurospora crassa*
PCA: Principal component analysis
*pcbAB*: Gene for δ-(L-α-aminoadipyl)-L-cysteinyl-D-valine synthetase
*pcbC*: Gene for isopenicillin N synthetase
*PclaeA*: Gene for methyl-transferase from *Penicillium chrysogenum* part of the velvet complex
*PcvelA*: Velvet gene from *P. chrysogenum*
*PcvelB*: Gene for Velvet subunit B from *P. chrysogenum*
*PcvelC*: Gene for Velvet subunit C from *P. chrysogenum*
*PcvosA*: Gene for spore development regulator VosA from *P. chrysogenum*
RAVEN: Reconstruction, analysis and visualization of metabolic networks
RT-qPCR: Reverse-transcription quantitative polymerase chain reaction
SNP: Single nucleotide polymorphism
TCA: Tricarboxylic acid
*Velvet*: Gene for subunit A of the Velvet complex
