# Supplementary figures and images for: Transcriptome analysis of the two unrelated fungal β-lactam producers Acremonium chrysogenum and Penicillium chrysogenum: Velvet-regulated genes are major targets during conventional strain improvement programs
Source: BMC Genomics. 2017 Mar 31;18:272. doi: 10.1186/s12864-017-3663-0 (PMC5374653; doi:10.1186/s12864-017-3663-0)

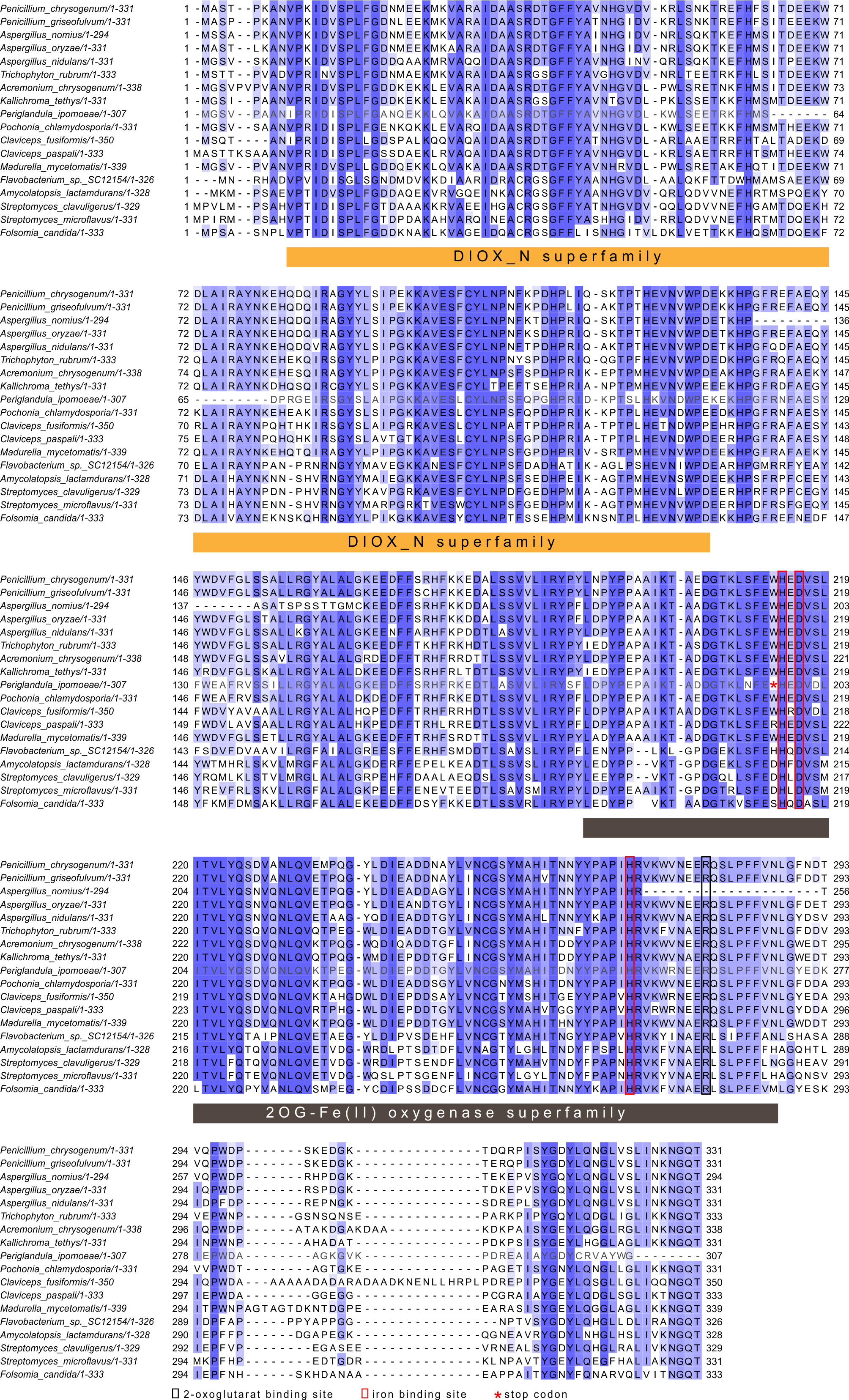

Supplement: Supplementary file 1 — Alignment of the amio acid sequence of various PcbC orthologs. Predicted protein domains are indicated by colored boxes. Substrate and cofactor binding sites are marked by black or red squares. The premature stop codon in the pcbC ortholog from P. ipomoeae is indicated by a red asterisk. (TIF 3780 kb) [file 12864_2017_3663_MOESM1_ESM.tif]

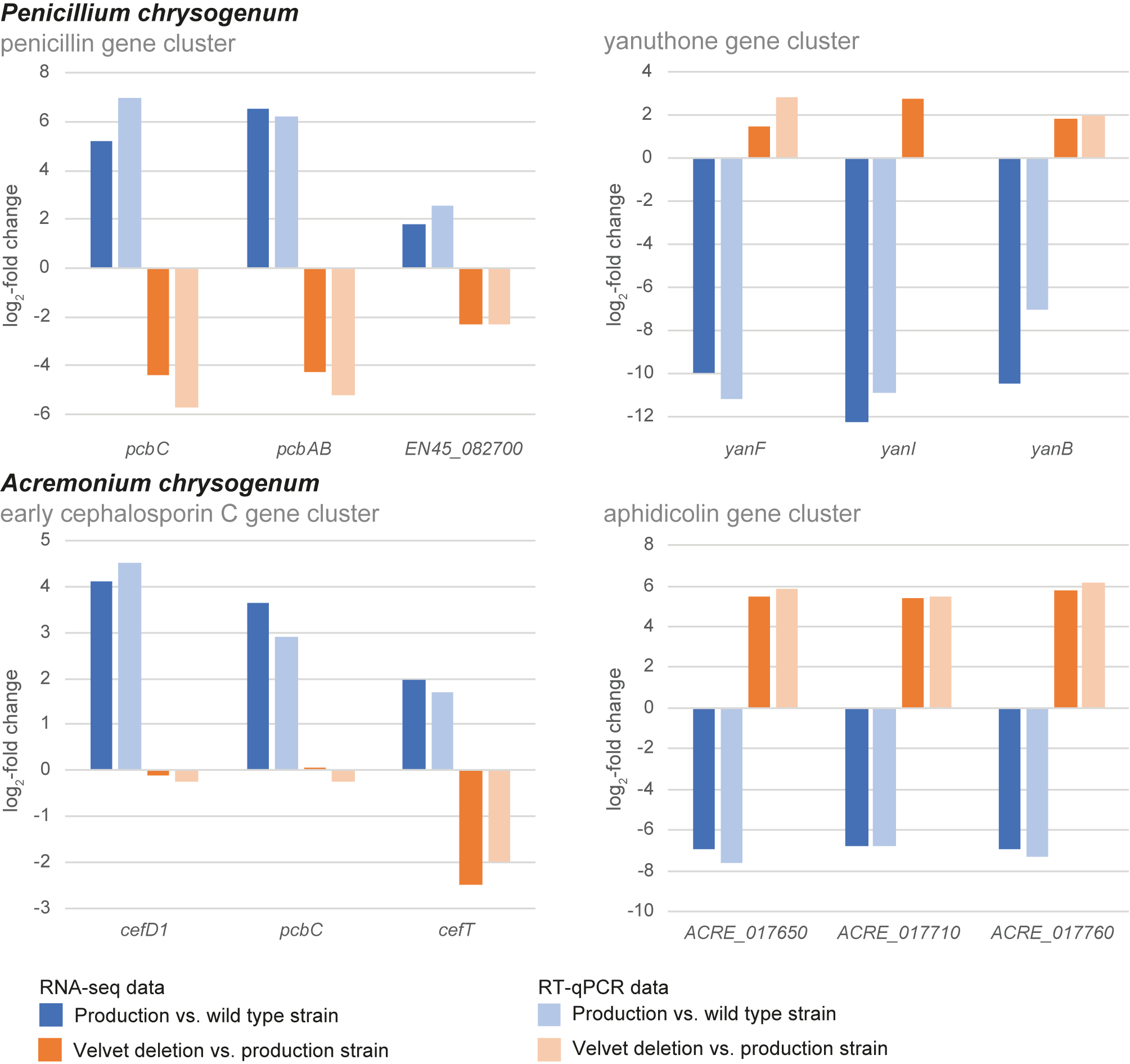

Supplement: Supplementary file 3 — Validation of RNA-seq data by RT-qPCRs. Expression levels of selected genes within the penicillin and yanuthone gene cluster from P. chrysogenum and within the early cephalosporin C and aphidicolin gene cluster from A. chrysogenum were confirmed by RT-qPCR. For each gene cluster, the change in expression (log2-fold) of three genes is shown in comparison to the results of the RNA-seq analyses. (TIF 612 kb) [file 12864_2017_3663_MOESM3_ESM.tif]

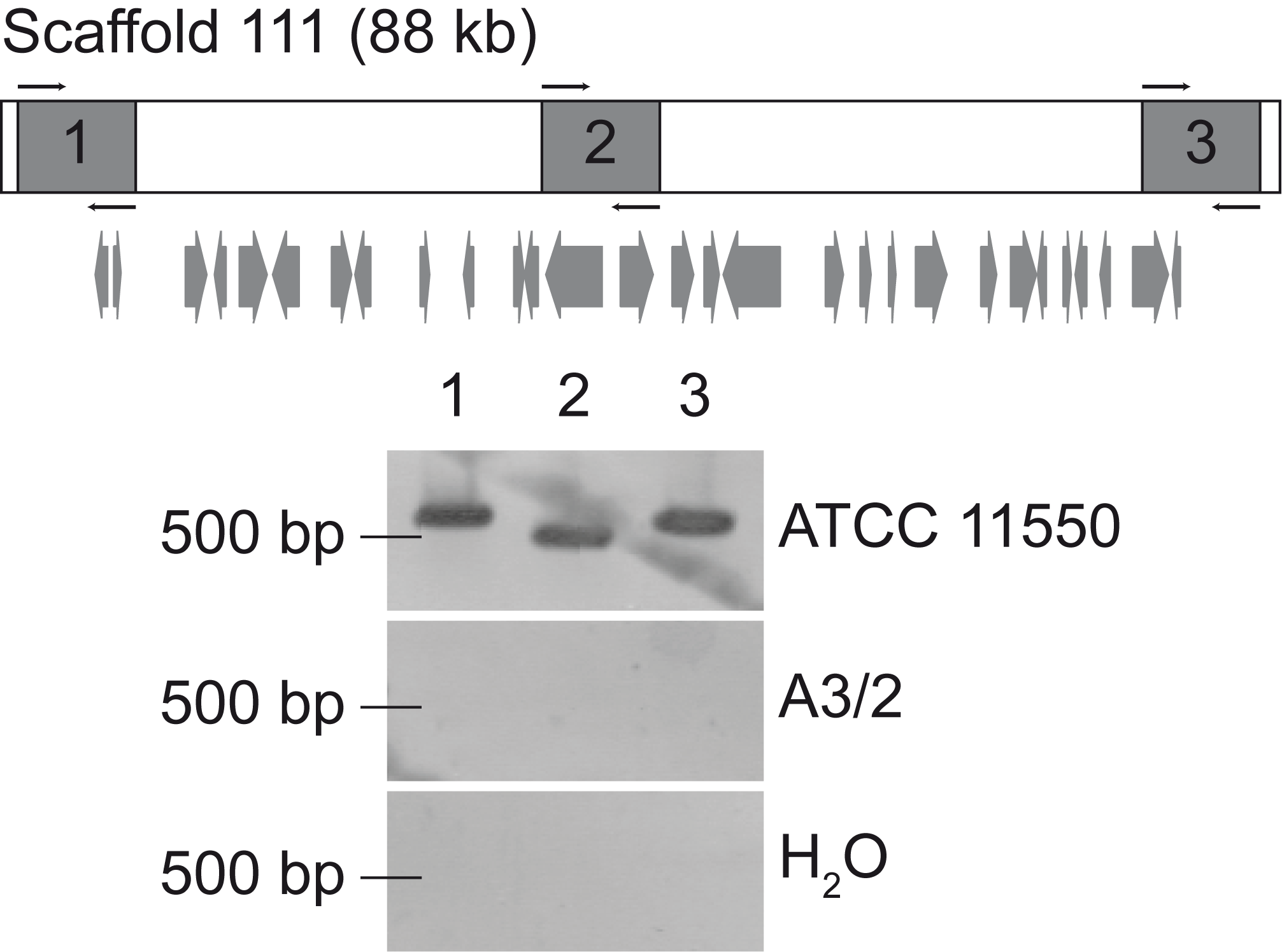

Supplement: Supplementary file 4 — PCR amplification of regions across scaffold 111 from A. chrysogenum. The presence and absence of scaffold 111 within the genomic sequence of wild-type strain ATCC 11550 and industrial strain A3/2 was verified by PCR. Grey arrows indicate open reading frames. Black arrows mark primer binding sites for the three amplicons. (TIF 717 kb) [file 12864_2017_3663_MOESM4_ESM.tif]

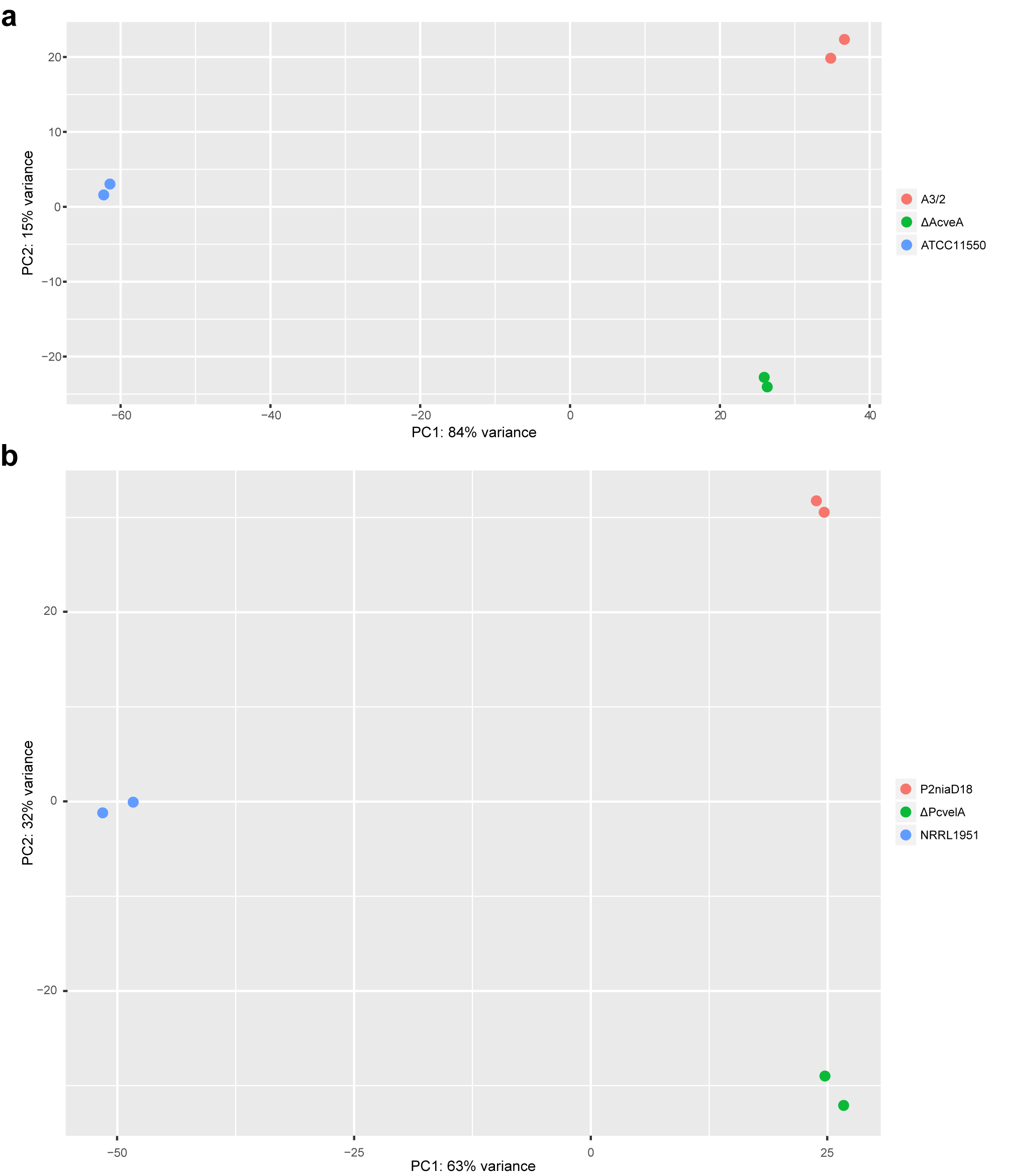

Supplement: Supplementary file 6 — Principal component analysis (PCA) from all included RNA-seq data sets. (a) PCA-plot for all RNA-seq data sets from P. chrysogenum. (b) PCA-plot for all RNA-seq data sets from A. chrysogenum. (TIF 523 kb) [file 12864_2017_3663_MOESM6_ESM.tif]
